# Frontiers in Rheumatoid Arthritis: Emerging Research and Unmet Needs in Pharmacologic Management

**DOI:** 10.3390/ph19020218

**Published:** 2026-01-27

**Authors:** Joshua J. Skydel, Betty Hsiao

**Affiliations:** Section of Rheumatology, Allergy & Immunology, Department of Internal Medicine, Yale School of Medicine, New Haven, CT 06520-8031, USA; betty.hsiao@yale.edu

**Keywords:** rheumatoid arthritis, DMARD, treat-to-target, difficult-to-treat, phenotyping, precision medicine, biomarkers, long-term safety, real-world evidence, personalized medicine

## Abstract

The management of rheumatoid arthritis (RA) has undergone several practice-defining evolutions, beginning with the approval of low-dose methotrexate and continuing through the introduction of numerous disease-modifying antirheumatic drugs (DMARDs). With increasing capability to target pro-inflammatory pathways, successive therapeutics have carried the promise of improved disease control for patients with RA; however, many patients still fail to meet treatment objectives, leading to the recognition of clinical phenotypes that remain therapeutically challenging under the current treat-to-target standard of care, including preclinical inflammatory arthritis, late-onset RA, and treatment-resistant RA. Precision medicine approaches are beginning to characterize the pathogenesis of RA in such populations, and to inform effective tailoring of DMARD therapy to individual patients. Simultaneously, observational data derived from clinical practice are increasingly being used to understand the risks and benefits of long-term DMARD therapy under real-world conditions of use, with registries and other observational sources confirming long-term effectiveness, revising safety profiles, and estimating the costs of treatment for approved therapies. Together, these strategies offer opportunities to address unmet needs in the care of patients with RA. In this review of peer-reviewed clinical and translational research in RA, we identify several clinical phenotypes that demonstrate inadequate response to guideline-directed therapy and review frontiers in clinical research in RA emerging over the last decade, highlighting the use of precision medicine and real-world evidence-based approaches to advance individualized, patient-centered care.

## 1. Introduction

Rheumatoid arthritis (RA) is the most common type of autoimmune arthritis, with an estimated prevalence of up to 1% of the global population [1]. Since the approval of low-dose methotrexate for the treatment of RA in 1988 [2], numerous disease-modifying antirheumatic drugs (DMARDs) have been approved for use in RA in the United States, including conventional synthetic DMARDs (csDMARDs), targeted synthetic DMARDs (tsDMARDs), and biological DMARDs (bDMARDs). The American College of Rheumatology (ACR) recently updated guidelines for the management of RA in 2021 [3], and consensus goals for pharmacotherapy have prioritized the reduction in disease activity. However, despite sustained research in RA management, a substantial proportion of patients with RA still do not achieve low disease activity or remission and/or lack a durable response to DMARD therapy. Several clinical phenotypes have been described, most notably “difficult-to-treat” RA, for which ongoing research is aimed at improving therapeutic strategies. The objective of this review is to synthesize frontiers in clinical and translational research in RA, emphasizing findings with the potential to refine the assessment of disease state and the optimization of clinical management using existing pharmacologic strategies.

## 2. Current Treatment Paradigms in RA

### 2.1. Guideline-Directed Treatment

In the United States, the standard of care for pharmacologic management in RA is largely informed by the 2021 ACR guidelines, developed by a voting panel comprising clinicians and patients, which strongly recommend initiation and optimization of methotrexate monotherapy for DMARD-naive patients with moderate or greater disease activity. Escalation of therapy by adding or switching to bDMARDs (e.g., tumor necrosis factor-alpha [TNF-a] inhibitor, T cell costimulatory inhibitor, interleukin [IL]-6 inhibitor, or anti-CD20 antibody therapy) or tsDMARDs (i.e., Janus kinase [JAK] inhibitors) is considered if disease control is not achieved with methotrexate alone [3]. DMARD-naïve patients with low disease activity at diagnosis may alternatively be treated with hydroxychloroquine or sulfasalazine instead of methotrexate. TNF-a inhibitors are widely accepted as first-line bDMARDs, although approximate therapeutic equivalence has been demonstrated across classes of United States Food and Drug Administration (FDA)-approved bDMARDs and tsDMARDs [4,5,6]. Combination therapy with csDMARDs represents another therapeutic strategy, most commonly triple therapy with methotrexate (or leflunomide), hydroxychloroquine, and sulfasalazine [7]; given tradeoffs between benefits (e.g., lower costs and less risk of adverse events) and drawbacks (e.g., high pill burden, longer lag time to take effect, and poor retention) [8], guidelines conditionally recommend escalation to b/tsDMARD over triple therapy, in most cases prioritizing response to therapy as quickly as possible. Glucocorticoid avoidance is recommended for most patients.

International guidelines for RA treatment are similar to current ACR guidelines, with some key differences. Methotrexate is universally preferred as first-line csDMARD therapy for patients, although ACR permits alternative csDMARD treatment in cases of low disease activity, with hydroxychloroquine preferred [9]. In contrast to ACR guidelines, which recommend against the routine short-term or long-term use of glucocorticoids when initiating DMARD therapy, the European League of Associations for Rheumatology (EULAR) and Asia Pacific League of Associations for Rheumatology (APLAR) allow for the use of tapering glucocorticoids at the time of diagnosis, for up to 3 or 6 months, respectively, as a bridge to therapeutic efficacy after initiation of DMARD therapy [9]. Amongst all guidelines, subsequent treatment escalation, including to b/tsDMARD therapy, is informed not only by disease activity but also by considering patient preferences, comorbidity, and risk for adverse events, as well as clinician experience, local DMARD availability, and cost (Table 1).

### 2.2. Treat-to-Target

Current guidelines recommend the adoption of a treat-to-target (T2T) strategy over usual care for RA management. This paradigm involves regular reassessment of disease activity with validated measures to guide adjustment and/or escalation of treatment, with the goal of achieving low disease activity or clinical remission [10]. Options for adjusting therapy include escalation of existing DMARD therapy (i.e., increasing dose or switching route of administration from oral to injection), combination therapy (i.e., adding DMARDs), or switching to a different class of DMARDs, with holistic reassessment of disease activity using composite measures of disease activity to drive treatment decisions. Although criteria for remission have been developed, an initial target of low disease activity may be preferred, as many patients may struggle to achieve strict targets due to comorbidities, treatment intolerance, or other patient-specific factors [11]. Overall, treatment goals should be individualized and based on clinical presentations as well as shared decision-making between patients and clinicians to ensure that the target is remission whenever feasible.

There is substantial evidence supporting the T2T approach, which demonstrates improved likelihood of remission, radiographic stability, and function and quality of life as compared to usual care [12]. Longitudinal analysis of patients with RA demonstrated an association of the T2T strategy with remission across disease activity indices [13]. Despite the proven benefits of T2T, adoption in real-world clinical settings is inconsistent. A systematic review of T2T literature has identified a variety of factors as potential barriers to uptake, including awareness of treatment goals, differences between clinician and patient perceptions of goal disease activity, perceived risks of escalating therapy, and resource constraints limiting possible DMARD choices [14]. Questions remain over what the optimal target is for individual patients [15], including preferred disease activity measures, or even who (i.e., clinicians or patients) is best positioned to evaluate longitudinal treatment outcomes [16]. Moreover, adherence is limited in real-world settings due to a variety of clinician, patient, and systemic factors [14,17], and optimal treatment targets have not been established [18]. Despite such implementation challenges, T2T remains the standard for care for RA.

## 3. Identifying Unmet Needs in the Management of RA

Despite the effectiveness of T2T as a treatment strategy for RA, many patients do not achieve remission in routine clinical practice [19], with a substantial proportion of patients having suboptimal responses to conventional guideline-directed therapies [20,21]. Several clinical phenotypes have been identified in which existing therapeutic strategies may be insufficient to fully address patient needs.

### 3.1. Treatment-Resistant RA

Recognition that a subset of patients fail to achieve treatment targets despite multiple lines of therapy led to the characterization of treatment-resistant RA phenotypes. Clinical heterogeneity among patients with treatment-resistant RA contributes to therapeutic and prognostic differences within this disease subpopulation (Table 2).

There is growing recognition of the difficult-to-treat (D2T) RA concept, defined as follows: (1) failure of 2 or more bDMARDs or tsDMARDs after initial treatment with csDMARDs per guidelines, (2) signs suggesting active or progressive disease, and (3) management of signs and symptoms considered problematic by the patient and treating clinician, such as intolerable side effects of treatment or complications of chronic glucocorticoid use [24]. Prevalence estimates for D2T RA vary significantly, with a recent review suggesting between 5.5% and 27.5% of patients may meet these criteria [25]. Numerous clinical features have been associated with D2T status, including younger age at diagnosis, female sex, seropositivity, and delayed treatment initiation [22]. Patients who progress to D2T status are more likely to have had high disease activity and radiographic damage at baseline [26,27]. Comorbidity relating to persistent inflammatory state, extra-articular disease, and the effects of long-term immunosuppressive therapy have also been described in D2T cohorts [28]. Pain and fatigue syndromes, including fibromyalgia, are more common amongst patients with D2T disease and are posited to contribute to suboptimal treatment outcomes [29]. DMARD failure in D2T RA may reflect some or all these elements, with a combination of biological processes (e.g., accumulated joint damage, immunogenicity, and inflammatory drivers such as obesity) and psychosocial factors (e.g., treatment adherence, comorbid anxiety and depression, limited coping with pain and disability) contributing to suboptimal treatment outcomes [30].

In addition, patients with treatment-resistant RA can be broadly characterized by an inflammatory state. Patients with persistent inflammatory refractory RA (PIRRA), despite multiple lines of therapy, may continue to present with polyarthritis or may resolve to one or a few joints with refractory synovitis [23]. In contrast, non-inflammatory refractory RA (NIRRA) is characterized by persistent arthralgia despite low swollen joint counts and inflammatory markers and is associated with coincident obesity and fibromyalgia [31]. The PIRRA and NIRRA phenotypes underscore the spectrum of potential causes underlying treatment failure in RA, ranging from refractory inflammation to accumulated damage associated with progressive arthritis to non-inflammatory comorbidities contributing to chronic pain. Accordingly, differentiation of patients by inflammatory state can inform goals for treatment, such as the use of intra-articular steroids or synovectomy referral for persistent synovitis in a solitary joint after DMARD therapy, or prioritization of the treatment of comorbidities in the absence of overt inflammation.

### 3.2. Late-Onset RA

Late-onset RA (LORA) refers to patients developing new inflammatory arthritis after the age of 60 years, with several distinctions between LORA and conventional presentations of RA, such as acute onset of symptoms, approximately equal sex distribution, and more frequent involvement of large proximal joints (e.g., shoulders and knees) [32]. Presentations with prominent bursal involvement resembling polymyalgia rheumatica, or related remitting seronegative symmetrical synovitis with pitting edema (RS3PE), have also been described [33]. Patients with LORA are less likely to be seropositive than younger patients [34]. Comorbid musculoskeletal conditions, including osteoarthritis and osteoporosis, are common and may confound assessment.

Treatment of LORA requires consideration of aging-related comorbidities that may contribute to increased treatment toxicity. Although response rates are comparable to patients with earlier-onset disease [35], LORA is associated with a greater incidence of adverse events during treatment, including declining physical function [36]. For patients aged 75 years and older, targeting low-disease activity may be more appropriate than remission, as it is imperative to balance the risks and benefits of treatment escalation in this population [37].

### 3.3. Preclinical and Early RA

Over time, increasing attention has been focused on identifying patients at risk of developing RA and developing preventative strategies for those with preclinical or early disease. The concept of preclinical RA represents the period of time during which patients begin to develop circulating rheumatoid factor (RF) and anti-citrullinated protein antibodies (ACPAs) that precede the development of inflammatory arthritis [38], with titers rising alongside increased systemic inflammation and early joint symptoms immediately prior to the onset of sustained synovitis consistent with diagnosis [39]. Although genetic risk for RA may be conferred by factors such as the HLA-DRB1 shared epitope, several modifiable risk factors have also been associated with the development of these pathogenic antibodies, including cigarette smoking, occupational exposures, obesity, and vitamin D deficiency [40]. Microbiome dysregulation, in particular overrepresentation of *P. gingivalis* and associated periodontitis, has also been identified as a risk factor for progression to RA in ACPA-positive individuals [41,42]. Other implicated microorganisms, including *P. copri*, *S. didolesgii*, and *S. parasanguinis*, contribute to RA pathogenesis through a variety of mechanisms, including the production of citrullinated or RA-mimicking (i.e., presented on HLA molecules containing the shared epitope) antigens, as well as induction of metabolic changes contributing to increased inflammation [43]. Of note, while genetic and environmental factors are predictive of the risk of developing RA, and in some cases of future disease severity, their utility for guiding preventative efforts or selection of DMARD therapy has not been clarified [44,45,46].

The association between palindromic rheumatism (PR) and RA has offered potential insights into the pathogenesis of RA. PR is an episodic arthritis characterized by flares of inflammatory arthritis that may last for days, resolving without incurring irreversible joint damage. Similarities in autoantibody profiles and patterns of joint involvement have been noted in PR and RA [47], with imaging studies that suggest that PR flares are characterized by increased extracapsular inflammation, as opposed to the synovitis characteristic of RA [48]. Although the relationship of PR and RA remains unclear, historical reports note a high (>50%) rate of progression to RA, and PR is sometimes considered to exist on the spectrum of seropositive arthralgia with other precursors to clinical RA [47].

Numerous therapies have been evaluated for use in RA prevention, although the results have been inconsistent. A systematic review of clinical studies evaluating prevention strategies in individuals at high risk of developing RA, or with undifferentiated inflammatory arthritis, did not identify any DMARD-based strategies that successfully prevented the onset of disease, although treatment with abatacept or rituximab was associated with a delayed onset of RA by up to 18 months [49]. While early treatment with methotrexate does not prevent progression to clinical arthritis, long-term outcomes with regard to inflammation and functional status were improved [50]. Trials of glucocorticoids and other csDMARDs such as hydroxychloroquine have been less successful [51]. Lifestyle interventions, including physical activity, stress reduction, and avoidance of environmental irritants (e.g., cigarette smoke or occupational exposures), are posited to reduce the risk of developing RA, although most evidence is derived from observational sources with limited ability to determine causality [52]. While RA prevention strategies have generally failed to prevent the onset of disease thus far, data show promise for modifying disease trajectory, suggesting potential utility for specific cohorts such as patients at risk for developing severe disease.

## 4. RA Research Frontiers

**Figure 1:**
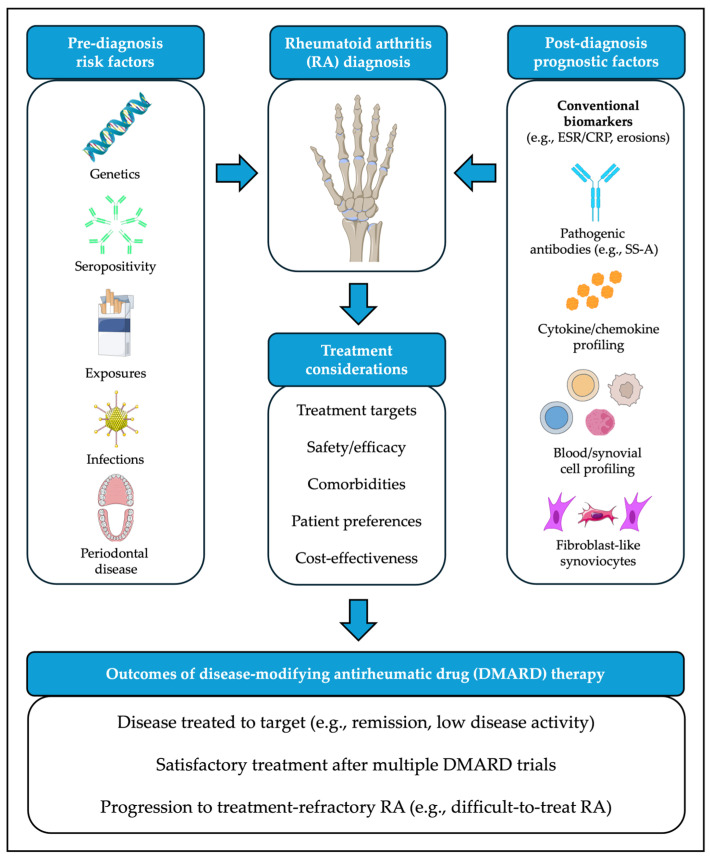
Clinical contributors to pathogenesis and treatment outcomes for rheumatoid arthritis (RA). Images adapted from bioicons (https://bioicons.com/, accessed on 20 January 2026), licensed under CC BY 3.0 (https://creativecommons.org/licenses/by/3.0/, accessed on 20 January 2026): “cigarette”, “teeth-gum-adult”, “fibroblast-4”, “adeno-virus” icons by Servier) or CC BY 4.0 (https://creativecommons.org/licenses/by/4.0/, accessed on 20 January 2026): “Posterior_view_of_bones_of_right_hand”, “B-DNA” icons by DBCLS; “Rubisco” icon by Margot Riggi; “B-cell-1”, “B-cell-4”, “cancer_cell” icons by El-Jayawant; “epithelial_cell”, “neutrophil”, “immuneglobuline_igG_blue”, “immuneglobuline_igG_green”, icons by Helicase 11).

Improved understanding of clinical phenotypes with suboptimal response to existing therapeutic strategies has fueled interest in developing precision medicine approaches to RA management. Molecular insights into the pathogenesis of the disease, as well as evidence drawn from large cohorts and other sources of real-world data, can aid in tailoring DMARD therapy at treatment initiation and/or at junctions when treatment changes are being considered (Figure 1).

### 4.1. Biomarkers

Biomarkers, a term encompassing a variety of entities derived from blood or other tissues usable to study biological processes, have long been utilized in the diagnosis and clinical evaluation of RA. Traditional laboratory evaluation in RA includes measurement of acute phase reactants (e.g., C-reactive protein, CRP, and erythrocyte sedimentation rate, ESR) for assessment of active inflammation, as well as characteristic autoantibodies (i.e., RF and ACPA) conventionally defining seropositivity. The history of RF and ACPA testing has been reviewed extensively in the literature. Summarized briefly, these autoantibodies, respectively, directed against the Fc region of immunoglobulin G and citrullinated proteins, both serve as useful biomarkers in the management of RA; ACPAs are commonly observed in early disease, and both are associated with higher disease activity, with RF predictive of extraarticular disease and ACPA commonly associated with more severe erosive arthritis (and, conversely, improved response to DMARD therapy) [53]. While not necessary for RA diagnosis, both autoantibodies, when present, can aid in risk stratification among patients. Only recently have additional biomarkers offered opportunities to further characterize individual patients and to inform the selection of therapy. While numerous RA biomarkers remain under varying stages of development [54], we have highlighted several that have shown promise for clinical implementation.

#### 4.1.1. Autoantibodies

In addition to RF and ACPAs, patients with RA may present with additional autoantibodies against proteins that have undergone non-citrullination post-translational modifications, including anti-carbamylated protein, anti-acetylated protein, and malondialdehyde-acetaldehyde antibodies observed in early clinical disease [55]. Antibodies against native proteins, including PTX3, DUSP11, and PAD4, have also been identified [56]. These more recently described autoantibodies frequently correlate with the development of conventional ACPAs and can be associated with radiographic progression. Although highly specific, they are less sensitive and not yet widely adopted in routine clinical practice.

Further serological testing may also enhance patient assessment. Most notably, while up to 7.5% of patients with RA demonstrate clinical evidence of co-occurring (“secondary”) Sjogren’s disease, rates of positivity for antibodies to SS-A are substantially higher, being noted in up to 15% of patients [57]. Secondary Sjogren’s disease is associated with higher RA disease activity [58,59,60] and is more commonly identified amongst patients with D2T-RA [61]. Anti-SS-A antibodies, even in the absence of sicca or other Sjogren’s disease manifestations, are associated with reduced response to methotrexate and TNF inhibitors [62,63], highlighting the potential need for alternative first-line DMARDs. Specifically, hydroxychloroquine and rituximab, both commonly used in the management of primary Sjogren’s disease, may offer theoretical benefit for such patients [57,58,59], although evidence from long-term studies in RA patients with positive anti-SS-A antibodies without overt Sjogren’s disease is lacking. Small studies have also shown beneficial effects of tocilizumab and abatacept for patients with RA with positive anti-SS-A antibodies not responding to TNF inhibition [58,64], with the latter also ameliorating symptoms of secondary Sjogren’s disease [65]. Regarding JAK inhibitors, the known association between Sjogren’s disease and lymphoma suggests careful consideration must be taken when selecting therapies to minimize malignancy risk. In the clinical development program for tofacitinib, incident lymphoma was observed amongst participants, with numerically greater rates of concomitant Sjogren’s disease in those developing malignancy, underscoring the need for careful monitoring in this population [66]. Additional cardiovascular comorbidities and serologies (e.g., antiphospholipid antibodies) are not only associated with an increased risk of treatment complications but may also give pause to consideration for treatment with JAK inhibitors.

While the presence of anti-SS-A antibodies in RA patients may aid prognostication and inform early treatment with non-TNF bDMARDs, their utility as a biomarker for RA remains incompletely understood. Although testing is readily available in the US and other developed nations, global differences in accessibility and cost are likely to persist. The interpretation of results also requires nuance; there are no established standards for the interpretation of titers, with major international RA guidelines not addressing the clinical relevance of anti-SS-A antibodies, and seropositivity does not differentiate between patients with and without secondary Sjogren’s disease or other overlapping connective tissue diseases. Finally, the relatively low prevalence of anti-SS-A antibodies in the overall RA population suggests that serostatus is unlikely to completely capture patients at risk of severe disease or failure of traditional treatment paradigms, with associations between seropositivity and other prognostic factors, such as D2T status, remaining incompletely understood. Further research is required to fully clarify the significance of anti-SS-A antibody testing for the clinical management of RA.

#### 4.1.2. Cytokine Profiles

Deconvolution of convergent pathways in the RA inflammatory cascade offers another approach to guide treatment decisions, with TNF-a, IL-1, IL-6, and other pro-inflammatory cytokines well-described as key drivers in the pathogenesis of RA [67]. Novel disease activity scores derived from cytokine profiling correlate with conventional measures and offer a more nuanced insight into inflammation than ESR or CRP [68]. Despite the increased sensitivity of cytokine-based testing, its utility in disease management for individual patients is poorly understood. For example, treatment with methotrexate is associated with reductions in TNF-a, IL-17, and IFN gamma [69], but bDMARD treatment results in less consistent cytokine responses [70]. Discovering how to target dominant pro-inflammatory pathways remains a goal of cytokine-based molecular phenotyping, especially for patients with D2T. For a subset of patients, profiling may also support combination therapy; in small studies, the addition of rituximab or anti-IL-17 to TNF inhibitor therapy showed promise as a therapeutic strategy for treatment-resistant patients, although the safety of dual bDMARDs requires further investigation [71]. In the future, cytokine profiling may offer clinicians additional insight into selecting between approved therapeutic mechanisms, with serial testing providing early evidence of secondary treatment failure to anticipate the need to change therapy.

### 4.2. Cellular Profiling

Characterization of cell types driving RA pathogenesis has focused on the synovium and peripheral blood, providing insights into disease mechanisms and potential differences in therapeutic response.

#### 4.2.1. Synovium

Translational research indicates that features of the synovial microenvironment may predict disease trajectory. Spatial transcriptomics have revealed tissue-resident macrophages expressing the cell surface receptor LYVE1 as critical to synovial homeostasis; these macrophages are lost in early RA, but successful treatment with csDMARDs is associated with restoration of macrophage networks [72]. Their role in more advanced disease remains unknown. In addition to macrophages, the synovial cell repertoire includes substantial populations of T cells, fibroblasts, and myeloid cells, among which increased T cells, in conjunction with fibroblasts, are most commonly associated with seropositivity and higher baseline disease activity [73]. The composition of synovial cell populations varies independently of patient factors such as age and sex but is correlated with current therapy, suggesting DMARDs may influence the synovial microenvironment. By describing cellular changes developing in the setting of therapy, such analyses suggest explanations for primary and secondary treatment failure and potential therapeutic avenues based on dominant synovial phenotypes after initial treatment [74]. Through gene expression and histological analyses, four synovial phenotypes for RA have been characterized, each with distinct clinical implications. Patients with the myeloid phenotype were more likely to respond to TNF inhibitor therapy, whereas those with the lymphoid phenotype had better responses to tocilizumab; low inflammatory (pauci-immune) and fibroid phenotypes, characterized by minimal or low evidence of pro-inflammatory pathway activation, tended to demonstrate lower response rates to DMARD therapy [75]. Fibroblast-like synoviocytes (FLS) are recognized as a key driver in chronic inflammation in RA and are often associated with seronegativity and D2T status; while there is interest in developing FLS-targeted medications, such therapeutic strategies remain an unmet need [76].

#### 4.2.2. Peripheral Blood

The accessibility of circulating immune cells has also facilitated investigation into their roles in systemic inflammation. Immunophenotyping of peripheral blood cells offers an alternative to clinical phenotyping or classical serologies to classify patients with RA. Notably, clustering patients by T cell and B cell abundance yields distinct clusters predicting differential responses to DMARD classes; treatment with the putative bDMARD or tsDMARD of choice is expected to provide the most beneficial results, including higher rates of low disease activity or remission than other agents [77]. Other studies have further characterized T cells in RA, with seropositive patients demonstrating increased dysregulation of Th17 and regulatory T (Treg) cells as compared to seronegative patients, a pathophysiologic process potentially mediated by IL-4 and associated with increased systemic inflammation [78]. T cell subsetting is under investigation for potential use in predicting response to specific therapies such as tocilizumab and abatacept [79]. In parallel, prognostic and therapeutic roles of autoreactive B cells [80,81], NK cells [82], circulating macrophages [83], and additional cell types continue to emerge. Finally, clonal hematopoiesis is increasingly recognized as contributing to immune dysregulation, not only in aging but also in RA. A large Finnish cohort study identified unique clonal pathways for seropositive patients, characterized by *DNMT3A* mutations associated with higher disease activity, as well as seronegative patients, who have an increased presence of *TET2* mutations thought to be implicated in pro-inflammatory signaling via IL-1β [84]. Despite observed associations, the mechanistic contribution of clonal hematopoiesis to systemic inflammation in RA remains incompletely clarified.

#### 4.2.3. Chemokines and Other Proteins

Increased expression of the B cell signaling molecule CXCL13 has been observed in patients with early RA, regardless of seropositivity, who do not respond to methotrexate, suggesting that earlier treatment with bDMARD or tsDMARD therapy may be warranted [85]. Likewise, upregulation of the vascular homeostasis protein ANGPTL4 has also been identified as a moderator of erosive disease via TNF-related pathways [86] and is being explored as a therapeutic target. However, not all efforts have led to the development of novel therapies. CCR5, a chemokine receptor expressed by synoviocytes, has been implicated in pro-inflammatory pathways via the activation of Th1 cells [87], but antagonizing therapeutic antibodies failed to demonstrate benefit [88]. To date, hundreds of putative biomarkers have been described, although few have defined roles in the evaluation or management of RA [89]. As new molecular signatures are identified, their utility as RA biomarkers or therapeutic targets must be clarified prior to clinical uptake.

### 4.3. Personalized DMARD Selection

A corollary of precision DMARD selection is the likelihood that patients may be exposed to therapeutics at earlier stages of disease, in novel combinations, and for prolonged periods of time. Currently, rates of DMARD discontinuation or switches are high, with median retention times of 24 to 36 months or less across DMARDs in most cohorts [90,91,92,93]. As we approach 30 years since the FDA approved the first bDMARD (etanercept, in 1998), clinicians will increasingly face decisions regarding treatment strategies for patients who have been exposed to decades of immunosuppressive therapy (Table 3). In addition to therapeutic safety monitoring, understanding the real-world effectiveness of b/tsDMARDs is essential to pharmacologic selection over long disease trajectories.

#### 4.3.1. Real-World Safety of Biologic and Targeted Synthetic DMARDs

Clinicians seeking to escalate DMARD therapy beyond methotrexate must navigate safety profiles arising from randomized trials, observational studies, analyses derived from administrative claims data, and other sources. Over time, large registries and population-based studies have refined assessments of b/tsDMARD safety, with several themes arising under real-world conditions.

Although a serious consideration, infection risk is comparable for most approved RA therapies, with fewer than 10 serious infections per 100 treatment years [95,96]. For TNF inhibitor therapies, risk appears to decline after the initial 6–12 months of treatment [97,98], a pattern that has been extrapolated for other therapeutic mechanisms, including rituximab [99]. While close monitoring for infection is warranted, evidence to date does not suggest that infection risk precludes long-term treatment for most patients with RA.

Malignancy remains a concern for patients exposed to prolonged immunosuppression, especially when increased systemic inflammation and advancing age are considered as additional risk factors. Population-based studies have reassuringly demonstrated no increased risk of malignancy overall for long-term TNF inhibition, anti-IL-6, or anti-CD20 therapy as compared to b/tsDMARD-naïve patients or age-matched controls [100]. Given the biological role of CTLA4, concern persists over abatacept’s effects on anti-tumoral T cell immunity; in a recent meta-analysis, pooled data from observational studies supported increased risk of malignancy for abatacept [101], although postmarketing safety data reveals no greater risk of malignancy for abatacept compared to other therapeutic mechanisms [102]. Studies on JAK inhibitors have inconsistently associated use with increased risk of malignancy [103], notably non-melanoma skin cancer [104], and decreased risk of other cancers, including those of GI origin [105]. Given persistent uncertainty over risk, cautious use is recommended, with careful surveillance for evidence of malignancy.

Real-world data also informs estimates of risk for characteristic adverse events first observed in the development of b/tsDMARDs. TNF inhibitor therapies remain contraindicated for patients with symptomatic heart failure [106], as well as for those who have or are at risk of developing multiple sclerosis [107,108], but are otherwise comparatively safe [109]. Although abatacept was initially suspected to exacerbate chronic obstructive pulmonary disease (COPD), this signal has not been observed in analyses of administrative data [110]. IL-6 inhibitors increase lipid levels, but their use has not been associated with increased risk of cardiovascular disease as compared to tsDMARDs [111,112]. Previous diverticulitis, on the other hand, remains a contraindication, with higher intestinal perforation rates than any other DMARD class corroborated in a large RA registry [113]. Despite findings in postapproval clinical trials that JAK inhibitors are associated with an increased risk of major adverse cardiovascular events (MACE) and venous thromboembolism (VTE) [114], only a class-wide increased risk of VTE has been consistently identified in subsequent analyses [115]. Notably, rates of MACE have proven comparable to those of TNF inhibitors [116], even for patients with a history of cardiovascular disease [117,118], raising uncertainty over the contribution of DMARD therapy to risk independent of that conferred by the hyperinflammatory state of RA, a risk that is only partially abrogated by effective treatment. Finally, other than rare reports of progressive multifocal leukoencephalopathy associated with rituximab, estimated to occur at a rate of 2.6 cases per 100,000 treated patients with RA [119], long-term use is generally well tolerated and is not associated with increased risk of infection [120,121].

An important caveat to the safety of b/tsDMARDs must be reserved for elderly patients with RA, including the LORA subpopulation. Major international guidelines do not differentiate between elderly and non-elderly patients with regard to treatment initiation or goals, including T2T [3,10]. However, patients with LORA are less likely to be started on DMARD therapy, with an analysis of Medicare beneficiaries estimating less than one-third of patients newly diagnosed with RA receive a DMARD [122]. Elderly patients receiving DMARD therapy are likely at greater risk of infection, as well as herpes zoster reactivation, compared to younger patients; other adverse events, including malignancies and cardiovascular disease, have been inconsistently demonstrated across studies [123]. Comorbidities associated with advancing age, such as renal insufficiency or cardiovascular disease, complicate therapy planning via narrowed therapeutic windows and perceived risk of adverse events, while concerns over adherence to complex regimens by elderly patients with mechanical limitations or cognitive impairment also contribute to prescriber hesitancy [124]. Patient perceptions of disease burden and expected benefits of treatment are likely to contribute to under-treatment as well, based on the de-prioritization of RA treatment as compared to other comorbidities by many elderly patients [125]. Despite perceived risks, elderly patients can be effectively and longitudinally treated with DMARDs of all classes [126,127], suggesting that safety concerns should not preclude treatment and rather can be considered on a personalized basis.

Although special considerations may be required for at-risk populations, real-world analyses have largely confirmed the tolerability of b/tsDMARDs. However, clinicians must weigh individual patient factors against continuously evolving clinical data when making treatment decisions.

#### 4.3.2. Anticipating Clinical Trajectories for Patients Treated with b/tsDMARDs

As conventional clinical trials only provide information on the outcomes of treatment over relatively short time periods, additional sources of clinical evidence are critical to understanding responses to treatment over intervals that more accurately reflect the natural course of RA. Clinical data derived from multiple observational sources now provide opportunities to evaluate b/tsDMARD performance in real-world settings.

Rheumatology registries have existed since the mid-twentieth century, yielding invaluable insights into the epidemiology of RA, comorbidities, and treatment outcomes [128]. Their presence at the forefront of computerized health information systems has allowed registries to capture increasingly granular data, facilitating therapeutic assessment via target trials and other novel designs. Registry-based research efforts continue globally to this day. For example, the “JAK-pot” collaboration leverages data from tens of thousands of b/tsDMARD treatment courses to explore the safety and comparative effectiveness of JAK inhibitors across clinical settings [129,130], including for patients with D2T disease. Although registry analyses require careful consideration of the generalizability of findings beyond enrolled populations, these curated sources of real-world data can provide insights into the selection and sequencing of DMARD therapy [131].

In addition to registries, large-scale observational studies increasingly answer clinical questions in RA, driven by the availability of clinical documentation and expanded testing for secondary analyses [132]. Efforts such as the Rheumatoid Arthritis Real-world Cohort Study in China (ReALSA) aim to collect data on patients longitudinally, correlating clinical, laboratory, imaging, and pathological data to facilitate evolving analyses of real-world patients with RA [133]. Observational studies can also support therapeutic strategies, such as improved response rates with combined bDMARDs and csDMARDs [134], although few studies have demonstrated a consistent superior benefit of one therapeutic strategy over others [135]. In one notable exception, the Kyoto University Rheumatoid Arthritis Management Alliance (KURAMA) cohort documented improvement in functional outcomes associated with increasing b/tsDMARD use over 10 years of follow-up; researchers observed more rapid improvement in disease activity with TNF inhibitors, but greater sustained responses to IL-6 inhibitors, addressing a knowledge gap left by the absence of comparative efficacy trials with the potential to inform clinical decision-making [136]. The development of biobanks can extend the reach of such studies, enabling reanalysis of biological specimens in light of future technologies and validating translational insights into RA pathophysiology [73,137].

#### 4.3.3. Outcome Measures

As observational studies have propagated, standardization of rheumatology outcomes assessments spurred by groups including ACR and the International Consortium for Health Outcomes Measurement (ICHOM) can facilitate meta-analysis of clinical outcomes across practice settings [138,139]. Others, including Outcome Measures in Rheumatology (OMERACT), have sought to describe and standardize outcome measures used in observational studies directly [140]. Given the marked heterogeneity of RA, no consensus has been reached on ideal outcome measures for the evaluation of RA, although the Clinical and Simplified Disease Activity Indices (CDAI/SDAI) and Disease Activity Score-28 (DAS28) are commonly used. Patient-reported outcome measures such as the Health Assessment Questionnaire (HAQ), Patient-Reported Outcomes Measurement Information System (PROMIS) score, and others also inform assessment by providing patient perspectives on function and disability [139]. As observational sources of data continue to develop, standardized outcome assessments are necessary to extend clinical trial findings and inform RA management beyond conventional drug development timelines.

#### 4.3.4. Cost-Effectiveness

Cost-effectiveness represents another means of informing DMARD selection. Systematic reviews of cost-effectiveness for RA management have identified substantial heterogeneity among analyses, including in which DMARDs are included in comparisons, methodology for defining effective care in observational data sources, and thresholds for cost-effectiveness [141]. However, it can be generally stated that b/tsDMARD therapies cost more than csDMARD-based management strategies, with costs associated with one year of effective treatment frequently eclipsing USD 100,000 in the United States [142]. Specific cost estimates for DMARDs vary within and between countries based on complex factors, including regional differences in availability and market competition, insurance coverage and reimbursement structures for specialty medications, and, where applicable, national health technology assessments that inform reimbursement by payers. Accordingly, studies have drawn differing conclusions about the use of b/tsDMARDs in various practice settings [143,144,145,146]. Limited evidence supports the cost-effectiveness of b/tsDMARDs in the United States as compared to csDMARDs [147], with lower costs associated with switching between classes as compared to trialing multiple agents in the same class (conventionally, TNF inhibitors) [148]. Earlier treatment with JAK inhibitors has been specifically identified, in one study, as a cost-effective strategy [149]. Of note, cost-effectiveness is not a static concept, as drug prices change over time. The advent of biosimilars for many bDMARDs has begun to reduce costs associated with the treatment of RA [150,151], with further cost savings likely as barriers to biosimilar prescribing and dispensing are eliminated [152]. Although cost-effectiveness may influence coverage decisions for b/tsDMARDs, uncertainty over the relative cost-effectiveness of various therapies suggests that clinical decisions are likely to rely primarily on individual patient factors for the foreseeable future.

#### 4.3.5. Shared Decision Making

While significant emphasis has been placed on translational breakthroughs in the management of RA, research findings should be viewed in terms of their potential to support shared decision-making (SDM) between patients and clinicians. One of the overarching principles of RA management under the T2T framework is the prioritization of patient preference in treatment decisions through SDM [10]. Despite such recommendations, SDM frequently falls short in real-world practice [153]. Factors associated with poor SDM among patients with RA include low health literacy, limited English proficiency, older age, and lower trust in physicians [154], suggesting potential benefits for interventions reducing barriers to patients’ understanding treatment options and advocating for personal care preferences.

There is no single approach to SDM for the management of RA, although groups such as OMERACT have articulated ideals for SDM, including means of assessing the impact of interventions [155]. Within the field of rheumatology, SDM tools are most frequently created for use in RA [156], with examples including clinician education modules, decision aids, and structured dialog intended to elicit patient questions and values that inform preferences for treatment (Table 4) [156,157]. However, uptake remains inconsistent in rheumatology practice, due to factors including a lack of validation across practice settings, as well as time constraints in clinical care. Nonetheless, there is substantial ongoing research into new SDM approaches, with multimodal strategies to improve health literacy and elicit care preferences under investigation [158]. In the absence of a consensus approach, management decisions should be made with respect for patients’ expectations for treatment, risk tolerance for adverse events, medication burden, willingness to escalate or change therapy, and other personalized considerations. SDM should continue to be prioritized under precision medicine approaches, with clinicians providing decision support to patients by helping interpret the increasingly granular data available at the point of care. This approach ensures that care is truly individualized and that personal health information is used to inform patient choices, rather than preempt them.

## 5. Conclusions

In this review, we discuss several unmet needs in RA management and recent developments in precision medicine approaches to RA. Advances in clinical and translational RA research can be understood along several pathways; improved description of RA phenotypes and an expanding body of evidence supporting the safety and effectiveness of approved DMARDs are converging to guide clinicians in the selection of personalized treatment regimens. Further work is needed to develop effective strategies across the clinical spectrum, including prevention in individuals at risk of developing RA and management of treatment-resistant RA, to integrate novel biomarkers into routine practice, and to inform novel uses of DMARDs such as dual biologic therapy. The intersection of such research objectives is in and of itself a priority of translational research in RA, as new technologies capable of anticipating the trajectory of disease offer opportunities to intervene with therapies tailored to individual patients, with the goal of preventing onset, reducing pain or the accrual of joint damage, and minimizing the complications of DMARD therapy. Where current strategies may not sufficiently control RA for all patients, emerging technologies such as neuromodulation, transdermal drug delivery systems, nanomedicines, cellular therapy, and others offer promise. Moreover, the importance of non-pharmacologic interventions, including weight loss, smoking cessation, and ensuring oral health, should not be deemphasized as new technologies and pharmacologic strategies enter routine clinical practice.

There are several limitations to the present work that warrant consideration. This review focuses on research addressing unmet needs in the pharmacologic management of RA, with an emphasis on optimizing the use of existing therapies for the management. While we have highlighted numerous approaches to clinical and biochemical phenotyping, it is not possible to provide a comprehensive historical overview of clinical and translational research findings in RA, nor to address every research finding with potential translational value for clinical management. Of note, uptake of new technologies is likely to differ across clinical settings, based on factors including regulatory approval, local expertise, and cost, such that their true impact on RA management cannot be predicted from preliminary feasibility assessments. Moreover, we do not specifically address novel therapeutic mechanisms for which no pharmacologic agents are currently approved. As of January 2026, there are more than 500 pending or ongoing clinical trials in RA registered on ClinicalTrials.gov, suggesting the scope of ongoing clinical development for the disease [159]. In addition, we did not include discussion of non-pharmacologic management for RA, including vagus nerve stimulation, which was notably granted FDA approval in 2025. Such interventions may offer benefits beyond those of conventional pharmacological management but are unfortunately beyond the scope of this review. Finally, it should be acknowledged that real-world data must be interpreted within the context of the studies, registries, and populations from which it is derived; the translation of safety and effectiveness findings between clinical practice settings and between observational and interventional sources of clinical evidence remains largely unvalidated. Of note, there is no central listing of global RA registries, nor a consensus standard for data collection, limiting our ability to meta-analyze findings or comprehensively assess the strengths and limitations of registries as a source of clinical evidence in RA. This non-systematic review should be viewed as an overview of emerging research of clinical interest, rather than as an exhaustive taxonomy of clinical and translational research in RA.

As we mark 75 years since the Nobel Prize was awarded for the discovery of cortisone, first used for the treatment of RA, advances in biomedical science continue to offer promise for individuals living with RA whose needs are not fully met by the standard of care.

## Figures and Tables

**Table 1:** Pharmacologic management of rheumatoid arthritis (RA) recommended by major international guidelines [9].

Guideline Organization	Guideline Year	Preferred Pharmacologic Management			Suggested Role of Systemic Glucocorticoids
		First-Line	Second-Line	Third- and Subsequent Lines	
American College of Rheumatology (ACR)	2021	MTX preferred over SSZ, LEF, or HCQ	bDMARD (+/− MTX) or tsDMARD preferred over “triple therapy” (MTX, HCQ, SSZ)	Alternate bDMARD or tsDMARD	Avoid for most patients
Asia-Pacific League of Associations for Rheumatology (APLAR)	2018	MTX preferred over LEF or SSZ	Alternate csDMARD per local availability	Combination csDMARD therapy, bDMARD, or tsDMARD	Up to 3 months
European Alliance of Associations for Rheumatology (EULAR)	2022	MTX preferred over LEF or SSZ	Combination csDMARD therapy or bDMARD	Alternate bDMARD or tsDMARD	Up to 6 months

Abbreviations: MTX, methotrexate; SSZ, sulfasalazine; LEF, leflunomide; HCQ, hydroxychloroquine; bDMARD, biologic disease-modifying antirheumatic drug; tsDMARD, targeted synthetic disease-modifying antirheumatic drug; csDMARD, conventional synthetic disease-modifying antirheumatic drug.

**Table 2:** Defining treatment-refractory rheumatoid arthritis (RA).

**Difficult-to-treat (D2T) RA [22]**	
Definition	(1) Failure to two or more b/tsDMARDs (of differing mechanisms), after failure of csDMARD therapy. (2) Evidence of active or progressive disease. (3) Patient and/or clinician perception that RA management is problematic.
Clinical considerations	- Risk factors include younger age at onset, female sex, high baseline disease activity, and seropositivity. - Greater comorbidity and near-term mortality than the general RA population.
Treatment considerations	- Treat-to-target strategy with frequent reassessment. - Consideration of alternate or exacerbating diagnoses. - Limited evidence to support treatment with JAK inhibitors, IL-6 inhibitors; possible role for addition of IL-17 inhibitor therapy.
**Refractory RA [23]**	
Definitions	(1) PIRRA: Refractory mono-, oligo-, or polyarthritis after treatment with multiple DMARD therapies. (2) NIRRA: Symptomatic disease after treatment with multiple DMARD therapies, with minimal or no evidence of active inflammation.
Clinical considerations	- Variable seropositivity; may be associated with different patterns of joint involvement. - Partial DMARD efficacy leads to less accumulated damage than in historical patients, but erosions, osteoporosis, and cardiovascular disease remain common. - High burden of functional impairment and comorbidities, including pain syndromes (e.g., fibromyalgia).
Treatment considerations	- Ensure DMARD adherence, minimize rapid therapeutic cycling. - Local therapy for residual monoarthritis. - Recognition and treatment of comorbidities (e.g., obesity or depression), including through supportive care and lifestyle modification.

Abbreviations: RA, rheumatoid arthritis; D2T, difficult to treat; b/tsDMARD, biologic or targeted synthetic disease-modifying antirheumatic drug; csDMARD, conventional synthetic disease-modifying antirheumatic drug; JAK, Janus kinase; IL, interleukin; PIRRA, persistent inflammatory refractory rheumatoid arthritis; NIRRA, non-inflammatory refractory rheumatoid arthritis.

**Table 3:** Pharmacotherapy for the management of rheumatoid arthritis in the United States.

Therapeutics	Mechanism of Action	Type	Route of Administration	Approved for Use in RA [94]
Included in the 2021 ACR management guidelines				
Methotrexate	Dihydrofolate reductase inhibition	csDMARD	PO, SC	Yes
Sulfasalazine	Anti-inflammatory; broad immunosuppressive effects	csDMARD	PO	Yes
Hydroxychloroquine	Lysosome stabilization; inhibits antigen presentation	csDMARD	PO	Yes
Leflunomide	Dihydroorotate dehydrogenase inhibition	csDMARD	PO	Yes
Adalimumab, certolizumab, etanercept, golimumab, and infliximab	TNF-alpha inhibition	bDMARD	SC, IV	Yes
Sarilumab, tocilizumab	IL-6 inhibition	bDMARD	SC, IV	Yes
Abatacept	CTLA-4 costimulation inhibition	bDMARD	SC, IV	Yes
Bariticinib, tofacitinib, upadacitinib	JAK inhibition	tsDMARD	PO	Yes
Rituximab	Anti-CD20 antibody	bDMARD	IV	Yes
Not included in the 2021 ACR management guidelines, but used in clinical practice				
Steroids (e.g., prednisone, dexamethasone) ^1^	NF-κB inhibition; broad anti-inflammatory and immunosuppressive effects	Glucocorticoids	PO, IA, IV	Many
Anakinra	IL-1 inhibition	bDMARD	SC	Yes
Azathioprine	Antimetabolite (purine synthesis inhibition)	csDMARD	PO	Yes
Tacrolimus	Calcineurin inhibition	csDMARD	PO	No
Cyclosporine	Calcineurin inhibition	csDMARD	PO	Yes
Historical or rarely used therapies				
Salicylates (e.g., aspirin)	COX inhibition	NSAID	PO	Many
Penicillamine	Reduces T-cell activation	csDMARD	PO	Yes
Gold salts (e.g., auranofin, thiomalate, aurothioglucose)	Reduces T-cell activation; broad anti-inflammatory and immunosuppressive effects	csDMARD	PO, IM	Yes
NSAIDs (e.g., naproxen, ibuprofen) ^2^	COX inhibition	NSAID	PO	Many
Mycophenolate ^3^	Inosine monophosphate dehydrogenase inhibition	csDMARD	PO	No
Minocycline	MMP inhibition; broad anti-inflammatory effects	Antibiotic	PO	No

Abbreviations: RA, rheumatoid arthritis; ACR, American College of Rheumatology; bDMARD, biologic disease-modifying antirheumatic drug; tsDMARD, targeted synthetic disease-modifying antirheumatic drug; csDMARD, conventional synthetic disease-modifying antirheumatic drug; NSAID, non-steroidal anti-inflammatory drug; TNF, tumor necrosis factor; IL, interleukin; CTLA-4, cytotoxic T-lymphocyte associated protein 4; JAK, Janus kinase; CD20, cluster of differentiation 20; NF-κB, nuclear factor kappa-light-chain-enhancer of activated B cells; MMP, matrix metalloproteinase; COX, cyclooxygenase; PO, per os (“by mouth” or oral administration); SC, subcutaneous; IV, intravenous; IA, intraarticular; IM, intramuscular. Notes: ^1^ ACR recommends against the routine use of systemic steroid therapy for most patients with RA. ^2^ Non-disease-modifying therapy, but remains in use for symptomatic relief of arthritis. ^3^ Limited benefit for articular disease, but used in the management of extraarticular manifestations (e.g., interstitial lung disease).

**Table 4:** Example shared decision-making (SDM) tools developed for use in patients with rheumatoid arthritis (RA) [157].

Type of SDM Tool	Description	Number of Publications Describing Use in RA	Reported Use in Relation to Clinical Care	Example(s)
Patient education	Provide information (e.g., about disease or medications) without eliciting patient preferences.	4	Before, during, after	- Paper-based summaries of prescribing information for DMARD therapies, provided in multiple languages. - Multimedia providing information about DMARD therapies for patients with low health literacy.
Decision aid	Provide information with additional resources to clarify patient preferences.	9	Before, during, after	- Information and guiding questions for patients considering the addition of a TNF inhibitor to methotrexate. - Education on RA natural history, followed by guiding questions to choose between bDMARD therapies. - Family planning education with questions to guide the timing of additional pregnancies.
Discrete choice experiment	Identify patient preferences by selecting between choices (e.g., medications).	1	During	- Select between DMARD therapies based on head-to-head comparisons of risk-benefit profiles and other characteristics.
Conjoint analysis	Identify patient preferences by eliciting the most important values (e.g., medication safety and cost, etc.)	1	During	- Elicit choices for changes to DMARD therapy based on relative weighting of potential risks, benefits, mechanism of action, route of administration, and cost.

Abbreviations: SDM, shared decision making; RA, rheumatoid arthritis; DMARD, disease-modifying antirheumatic drug; TNF, tumor necrosis factor; bDMARD, biologic disease-modifying antirheumatic drug.

## Data Availability

No new data were created or analyzed in this study. Data sharing is not applicable to this article.

## Abbreviations

The following abbreviations are used in this manuscript:

RA	Rheumatoid arthritis
DMARD	Disease-modifying antirheumatic drug
ACR	American College of Rheumatology
TNF-a	Tumor necrosis factor-alpha
IL	Interleukin
CD20	Cluster of differentiation 20
JAK	Janus kinase
FDA	(United States) Food and Drug Administration
EULAR	European Association of Alliances for Rheumatology (formerly European League Against Rheumatism)
APLAR	Asia-Pacific League of Associations for Rheumatology
T2T	Treat-to-target
D2T	Difficult-to-treat
PIRRA	Persistent inflammatory refractory rheumatoid arthritis
NIRRA	Non-inflammatory refractory rheumatoid arthritis
LORA	Late-onset rheumatoid arthritis
RS3PE	Remitting seronegative symmetrical synovitis with pitting edema
RF	Rheumatoid factor
ACPA	Anti-citrullinated protein antibody
HLA	Human leukocyte antigen
PR	Palindromic rheumatism
PTX3	Pentraxin 3
DUSP11	Dual specificity phosphatase 11
PAD4	Peptidyl arginine deiminase type 4
SS-A	Sjögren syndrome-related antigen A
CRP	C-reactive protein
ESR	Erythrocyte sedimentation rate
IFN	Interferon
LYVE1	Lymphatic vessel endothelial hyaluronan receptor 1
FLS	Fibroblast-like synoviocyte
NK	Natural killer
DNMT3	DNA methyltransferase 3
TET2	Tet methylcytosine dioxygenase 2
CXCL13	C-X-C motif chemokine ligand 13
ANGPTL4	Angiopoietin-like 4
CCR5	C-C chemokine receptor type 5
CTLA4	Cytotoxic T-lymphocyte-associated protein 4
GI	Gastrointestinal
COPD	Chronic obstructive pulmonary disease
MACE	Major adverse cardiovascular events
VTE	Venous thromboembolism
ReALSA	Rheumatoid Arthritis Real-world Cohort Study in China
KURAMA	Kyoto University Rheumatoid Arthritis Management Alliance
ICHOM	International Consortium for Health Outcomes Measurement
OMERACT	Outcome Measures in Rheumatology
CDAI	Clinical Disease Activity Index
SDAI	Simplified Disease Activity Index
DAS28	Disease Activity Score-28
HAQ	Health Assessment Questionnaire
PROMIS	Patient-Reported Outcomes Measurement Information System
SDM	Shared decision-making
